# Does the HPV vaccination programme have implications for cervical screening programmes in the UK?^☆^

**DOI:** 10.1016/j.vaccine.2014.01.087

**Published:** 2014-04-01

**Authors:** Helen Beer, Sam Hibbitts, Sinead Brophy, M.A. Rahman, Jo Waller, Shantini Paranjothy

**Affiliations:** a Public Health Wales, Screening Division, Cervical Screening Wales, 18 Cathedral Road, Cardiff CF11 9LJ, Wales, UK; b School of Medicine, Cardiff University, Cardiff CF14 4XN, Wales, UK; c Health Information Research Unit, Swansea University, College of Medicine, Swansea SA2 8PP, Wales, UK; d Cancer Research UK Health Behaviour Research Centre, Research Department of Epidemiology and Public Health, UCL, Gower Street, London WC1E 6BT, UK; e Centre for Improvement in Population Health through E-records Research, Swansea University, College of Medicine, Swansea SA2 8PP, Wales, UK

## Abstract

In the UK, a national HPV immunisation programme was implemented in 2008 for girls aged 12–13 years. In addition a catch-up programme was implemented for older girls up to 18 years of age from 2009 to 2011, with an uptake rate of 49.4%. Information about future uptake of cervical screening according to vaccination statistics is important in order to understand the impact of the vaccination programme and implications for a national cervical screening programme. We analysed data on a cohort of women who had been offered the HPV vaccine in the catch-up programme and were invited for cervical screening between 2010 and 2012 in Wales (*n* = 30,882), in a record-linked database study, to describe the cervical screening uptake and clinical outcome according to HPV vaccination status.

In our cohort, 48.5% (*n* = 14,966) women had had HPV vaccination and 45.9% (*n* = 14,164) women attended for cervical screening. Women who were unvaccinated were less likely to attend cervical screening (adjusted OR 0.58; 95% CI (0.55, 0.61)). Of those who attended for screening, 13.9% of vaccinated women had abnormal cytology reported compared to 16.7% of women who were unvaccinated. Women who lived in areas with high levels of social deprivation were less likely to be vaccinated (Quintile 5 OR 0.48 95% CI (0.45, 0.52)) or attend cervical screening (Quintile 5 OR 0.70; 95% CI (0.65, 0.75)) compared to those who lived in the least deprived areas.

These data highlight the need for new strategies to address inequalities in cervical screening uptake and can inform further mathematical modelling work to clarify the impact of the HPV vaccination programme on future cervical cancer incidence.

## Highlights

• Women who did not take up the HPV vaccination were less likely to attend for cervical screening.
• HPV vaccinated women who attended cervical screening had the lowest proportion of cytological abnormalities detected.
• Social deprivation was the main factor-affecting uptake of both HPV vaccination and cervical screening.

## 1 Introduction

There are two commercially available Human Papillomavirus (HPV) vaccines licensed by the FDA for prevention of cervical cancer: Cervarix^®^ (GlaxoSmithKline) and Gardasil^®^ (Sanofi Pasteur MSD). Both vaccines prevent acquisition of HPV16 and 18 infections [1–5] responsible for approximately 70% of cervical cancers and they offer some cross protection against other oncogenic strains of HPV [6–10]. Clinical trial data has indicated that the vaccines are highly effective in preventing new cases of HPV16 and 18 associated diseases, with significantly lower rates of high grade Cervical Intraepithelial Neoplasia (CIN) and Adenocarcinoma in-situ diagnosed [11–15]. Prevention of cancer is more likely in women who receive the HPV vaccination prior to exposure to the virus [6,16].

In the UK, a national HPV vaccination programme using the bivalent vaccine, Cervarix^®^ was introduced in September 2008 in schools, with a recommended 3 doses administered to girls aged 12–13 years. A two-year catch-up vaccine arm was added for older girls who potentially would still benefit from the immune response induced by the HPV vaccine. Such a comprehensive national vaccination programme is expected to change the epidemiology of cervical cancer in the UK population. However, the impact of such a programme will depend on vaccine uptake, cervical screening uptake and the risk of exposure in women who are not vaccinated and not screened. If women who are unvaccinated choose not to attend for cervical screening, and have high risk of exposure to HPV, then the impact of the vaccination programme will be less than predicted, with potential to increase inequalities in cervical cancer incidence in the population.

In order to understand the likely impact of the HPV vaccination programme for cervical cancer incidence it is important to understand the screening behaviour of women according to whether or not they have been vaccinated. In this study we report the factors associated with HPV vaccination uptake, cervical screening uptake and clinical outcome according to HPV vaccination status for the first cohort of women who had been offered the HPV vaccine and were invited for cervical screening within a national population-based cervical screening programme in Wales.

## 2 Materials and methods

### 2.1 Study population

The study population comprised women who were born between 1 st September 1990 and 29th February 1992 who were resident in Wales on 1 st April 2012. These women would have been offered HPV vaccination as part of the catch-up campaign, and invited for routine cervical screening between 1st September 2010 and 29th February 2012 as they turned 20 years of age, or after moving into Wales.

### 2.2 Data sources

#### 2.2.1 Secure Anonymised Information Linkage databank

The Centre for Improvements in Population Health through e-Records (CIPHeR) has established the Secure Anonymised Information Linkage (SAIL) databank, which brings together and anonymously links a wide range of person-based data [17]. This databank includes existing routinely collected datasets such as the Welsh Demographic Service (all people registered with a Welsh or English General Practitioner), Cervical Screening Wales (CSW) (data from a population based national screening programme [18]) and the National Community Child Health Database (NCCHD) (child health records of children who since 1987 have been born, treated (including vaccination status) or resident in Wales [19]). Using these linked datasets we identified all women resident in Wales on 1st April 2012 within the cohort birth range, 1st September 1990 to 29th February 1992.

HPV vaccination data (number of doses and dates administered) were extracted from both the CSW and NCCHD databases and triangulated to give a complete vaccination history for the cohort of women. Data on cervical screening uptake and clinical outcome were obtained from the CSW databases. Data on birth characteristics of the women such as maternal age at birth, gestational age at birth and childhood vaccination status (for any childhood vaccinations as per the recommended schedule for immunisations in the UK) were extracted from the NCCHD. Data on quintile of social deprivation was based on Townsend score calculated using data from the 2001 Census, based on the woman's area of residence on April 1st 2012.

### 2.3 Statistical analyses

All analyses were carried out using SPSS v19. Univariate binary logistic regression was used to describe the association between each variable (quintile of social deprivation, maternal age at birth, gestational age at birth, childhood vaccination) and (i) HPV vaccination uptake, (ii) cervical screening uptake and (iii) cervical screening abnormality. Multivariate binary logistic regression was used to obtain odds ratios for the association between HPV vaccination uptake and cervical screening uptake, adjusted for the variables listed above.

Women were categorised as having been partially HPV vaccinated if only 1 or 2 of the recommended 3 doses were recorded, and fully HPV vaccinated if 3 or more doses were recorded.

Childhood vaccination was defined as any childhood vaccination recorded on the NCCHD database (excluding HPV vaccination). A cervical screening cytological abnormality was defined as a result of borderline changes, mild/moderate/severe dyskaryosis or worse. Results reported as inadequate or negative were considered not abnormal for this analysis.

Data were missing for some variables in the cohort: maternal age (29.7%); gestational age (33.9%); and childhood vaccinations (21.1%). We carried out a complete case analysis and analysis that included the missing data as a separate category. The results were similar in both models so we have presented the results with missing data as a separate category.

The analyses were restricted to cases with available social deprivation data based on the Townsend score for deprivation quintile [20], therefore excluded 12 women resident in Wales on 1st April 2012 for whom data on area of residence was missing.

## 3 Results

**Fig. 1:**
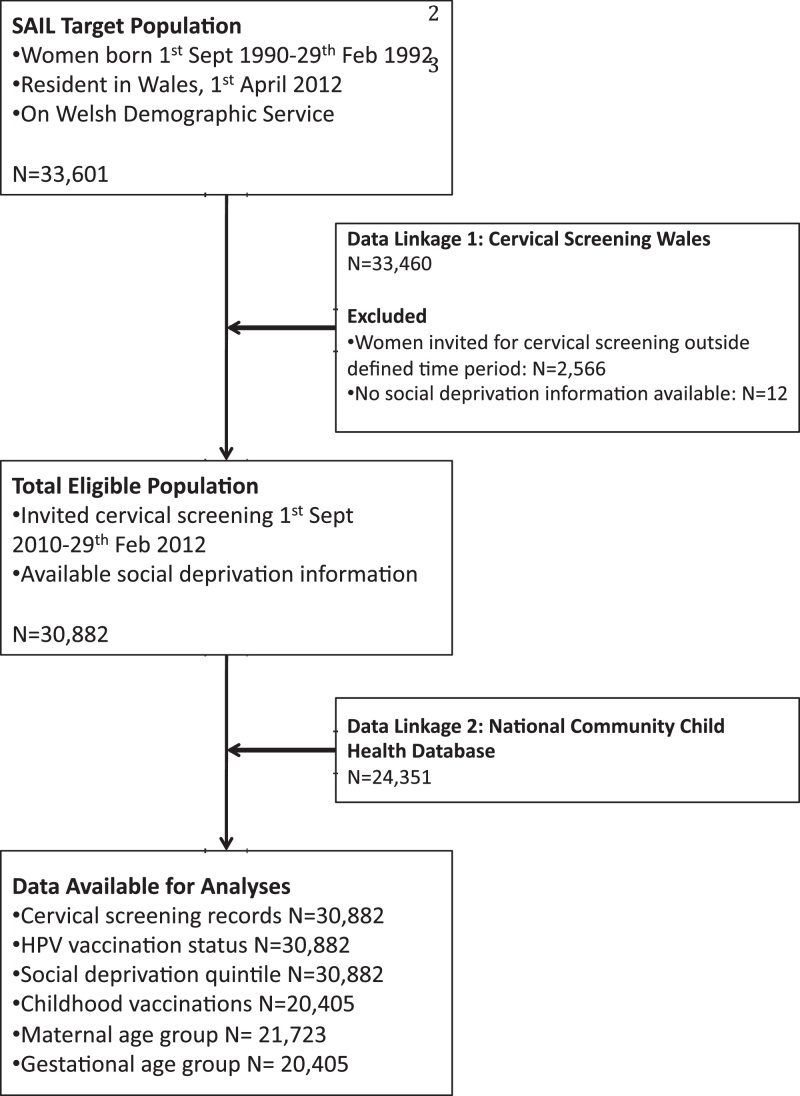
Study flow chart.

There were 33,601 women on the NHS AR for the study cohort and time period. Data were available for 30,882 women from the CSW and 24,351 women from the NCCHD (Fig. 1). 14,966/30,882 (48.5%) women had HPV partial or full vaccination and 14,164/30,882 (45.9%) women had attended for cervical screening. 2427/30,882 (7.9%) women had HPV partial vaccination and attended for cervical screening and 5579/30,882 (18.1%) women had HPV full vaccination and attended for cervical screening.

Table 1 describes the characteristics of women according to HPV vaccine uptake. HPV vaccination status was defined as (i) full HPV vaccination with 3 or more recorded doses (*n* = 10,109/30,882; 32.7%); (ii) partial HPV vaccination with 1–2 doses (*n* = 4857/30,882; 15.7%); (iii) not HPV vaccinated (*n* = 15,916/30,882; 51.5%).

There was a statistically significant relationship between uptake of the HPV vaccine and social deprivation quintile (Table 1). Women from the most affluent quintile (Quintile 1) were more likely to have had partial (19.2%) or full (39.5%) HPV vaccination. Conversely women from the most deprived quintile (Quintile 5) had the highest number of women that had not been HPV vaccinated and the lowest number of women with reported partial and full HPV vaccination (59.2%, 14.4% and 26.3%, respectively).

The highest proportion of women not vaccinated was observed for the groups with maternal age under 20 years and 20–24 years (55.4% and 48.7%, respectively) compared to groups whose mothers were older and this was statistically significant (OR 0.62; 95% CI (0.56, 0.68) and OR 0.80; 95% CI (0.75, 0.86), respectively). There was no clear relationship between gestational age and HPV vaccination.

Table 2 describes the uptake of cervical screening according to characteristics of women. There was a significant relationship between uptake of cervical screening and social deprivation score. Women from the most deprived areas (Quintile 5) were less likely to have attended for cervical screening than women from the least deprived areas (Quintile 1) (41.3% compared to 50.1%, respectively; univariate OR 0.69; 95% CI (0.65, 0.75)).

Women who were fully vaccinated were more likely to have attended for cervical screening than women who had not been vaccinated and this was statistically significant (55.2% compared to 38.7%, respectively, OR 0.51 95% CI (0.49, 0.54)). In women who had attended cervical screening, 8006/14,164 (56.5%) had received at least one dose of the HPV vaccine. In women who had not attended for cervical screening, 6960/16,718 (41.6%) had received at least one dose of the HPV vaccine.

Reported cervical screening cytological abnormalities in the study population are shown in Table 3. There was a clear relationship between HPV vaccination and cytological results with women attending cervical screening who had full HPV vaccination having the lowest proportion of abnormal cytology reported compared to those not vaccinated (OR 1.24; 95% CI (1.12, 1.37)). There was no relationship between reported cytological abnormality and social deprivation quintile, maternal age, gestational age or previous childhood vaccination.

Table 4 presents attendance for cervical screening and detection of abnormalities for women in each vaccination group, stratified by quintile of deprivation. Results indicate that HPV vaccination and social deprivation quintile are predictors of uptake of cervical screening but do not predict detection of abnormalities.

## 4 Discussion

This is the first UK study to investigate uptake of cervical screening following implementation of the HPV vaccination programme in the catch-up group. In contrast to concerns that vaccination would have a negative impact on a woman's decision to attend for cervical screening, uptake of the HPV vaccine was positively correlated to uptake of cervical screening. Social deprivation was the main factor affecting uptake of both the HPV vaccine and cervical screening, with the highest levels of non-participation observed in the most deprived quintile (59.2% unvaccinated and 58.7% unscreened compared with 41.3% and 49.9% in the least deprived quintile).

In women who attended for cervical screening, HPV vaccination had a protective effect with the lowest proportion of cytological abnormalities detected (86.1% normal cytology in fully vaccinated compared with 83.3% in the unvaccinated women; see Table 3). Although social deprivation affected uptake of both health services investigated, in this study population, social deprivation score was not associated with cytological result. The implementation of the HPV vaccination programme within schools has helped to reduce the impact of social deprivation on uptake of this health service with more than 80% uptake of all three doses of the HPV vaccine in girls aged 12–13 years [21].

The main strength of this study was the large sample size from an unselected population-based cohort utilizing record linkage of routinely collected data on HPV vaccinations and cervical screening. Quality of data, particularly the HPV vaccination history, was strengthened by the use of combined data from both the CSW and NCCHD datasets.

We are confident of the quality of the data used in this analysis as the HPV vaccination rates for this cohort are identical to published rates. The national statistics reported 32.8% of women had received all 3 doses of the vaccine in the birth cohorts 1st September 1990 to 21st August 1991 and 1st September 1992 to 31st August 1992 (PHW COVER Report 96 [22])

It is important to note that our results are based on analysis of women from the catch-up arm of the HPV vaccination programme. These women were older, and were not all in school and inequalities in coverage have been observed and reported [21]. Bowyer et al. quantitatively assessed the knowledge and awareness of HPV and the vaccine, amongst schoolgirls who had already been offered the HPV vaccine in the targeted UK vaccination programme [23]. In this cohort, knowledge about HPV infection was relatively low, and only 53.1% participants were aware that HPV could cause cervical cancer. Approximately half of the participants were aware that cervical screening was still required after HPV vaccination. In our data analyses, although the women studied were from the catch-up arm of the programme, we observed approximately half of the vaccinated cohort attending cervical screening (55.2%).

Analysis of factors potentially affecting uptake of health services available for primary cervical cancer prevention in the UK, highlighted that women who originate from more socially deprived areas are less likely to engage with the services available. Moreover, 9758/30,882 (31.6%) had neither attended for screening nor received the HPV vaccine. However, although social deprivation affected the initial engagement, once women engaged, at least in this age group, there was no significant difference in clinical outcome. Cervical cancer rates are higher in women from more socially deprived backgrounds [24]. However, data from our study suggests that this is a consequence of women from more socially deprived areas not engaging with the current primary cervical cancer prevention strategies in the UK.

In women offered HPV vaccination through the catch-up arm of the programme, this study shows a protective effect with a reduction in cytological abnormalities from 16.7% in unvaccinated women to 13.9% in vaccinated women. However, the level of abnormalities detected in the vaccinated women is still relatively high, potentially reflecting acquisition of the virus prior to vaccination. This data suggests that the catch-up arm of the vaccination programme has not had a substantial protective effect and a higher impact on cytological abnormalities is anticipated in the target group, who may not have been exposed to the virus prior to vaccination. Women who have chosen to receive the HPV vaccination and attend for cervical screening may be more health conscious, and this may be reflected in their sexual behaviours. It is therefore possible that they may be less likely to become infected with HPV, accounting for the reduction seen in the proportion of cytological abnormalities.

The findings reported here emphasise the need to promote further engagement with health services in more socially deprived areas with a focus on younger age groups to enhance the potential benefit of prevention programmes in early diagnosis and treatment long term. The HPV vaccination programme represents an ideal opportunity to convey the benefit of prevention programmes and reinforcement of this message is needed.

## 5 Conclusions

Uptake of HPV vaccination was positively correlated with uptake of cervical screening, and cytology results indicate that vaccination has a protective effect against an abnormal result. Women from more socially deprived areas engage less with cervical cancer prevention healthcare services. New strategies to enhance uptake of screening services need to be directed at young women with a focus on areas classified as socially deprived.

## Author contributions

SP and SH conceived of the study. HB, SB and MAR collected the data for the study. HB, SH and SP contributed to the analyses of the study and all authors contributed to the interpretation of results and the writing of this paper and have approved the final draft.

## Conflicts of interest statement

All authors declare no conflicts of interest that could have influenced this work.

## Figures and Tables

**Table 1:** HPV vaccination uptake according to characteristics of women in the cohort.

Variable	Number (% for each variable)		Fully HPV vaccinated (%)		Partially HPV vaccinated (%)		Not HPV vaccinated (%)		Univariate odds ratio (95% CI)		Adjusted odds ratio (95% CI)	
Quintile 1 (least deprived)	4093	(13.3)	1616	(39.5)	787	(19.2)	1690	(41.3)				
Quintile 2	4323	(14.0)	1680	(38.9)	708	(16.4)	1935	(44.8)	0.87	(0.80, 0.95)	0.90	(0.82, 0.98)
Quintile 3	5737	(18.6)	1993	(34.7)	901	(15.7)	2843	(49.6)	0.72	(0.66, 0.78)	0.83	(0.76, 0.90)
Quintile 4	6940	(22.5)	2241	(32.3)	1048	(15.1)	3651	(52.6)	0.63	(0.60, 0.69)	0.79	(0.73, 0.85)
Quintile 5 (most deprived)	9789	(31.7)	2579	(26.3)	1413	(14.4)	5797	(59.2)	0.48	(0.45, 0.52)	0.7	(0.62, 0.72)
Maternal age group 1 (25–29 years)	7576	(24.5)	2917	(38.5)	1378	(18.2)	3281	(43.3)				
Maternal age group 2 (under 20 years)	2163	(7.0)	572	(26.4)	393	(18.2)	1198	(55.4)	0.62	(0.56, 0.68)	0.65	(0.59, 0.71)
Maternal age group 3 (20–24 years)	6313	(20.4)	2089	(33.1)	1149	(18.2)	3075	(48.7)	0.80	(0.75, 0.86)	0.83	(0.78, 0.89)
Maternal age group 4 (30–34 years)	4093	(13.3)	1723	(42.1)	698	(17.1)	1672	(40.9)	1.11	(1.02, 1.20)	1.09	(1.01, 1.18)
Maternal age group 5 (35+ years)	1578	(5.1)	605	(38.3)	282	(17.9)	691	(43.8)	0.98	(0.88, 1.09)	0.97	(0.87, 1.09)
Maternal age group 6 (missing data)	9159	(29.7)	2203	(24.1)	957	(10.4)	5999	(65.5)	0.40	(0.38, 0.43)	1.09	(0.96, 1.24)
Gestational age group 1 (37+ weeks)	19,187	(62.1)	7115	(37.1)	3414	(17.8)	8658	(45.1)				
Gestational age group 2 (under 36 weeks)	1218	(3.9)	435	(35.7)	210	(17.2)	573	(47.0)	0.93	(0.82, 1.04)	0.95	(0.84, 1.07)
Gestational age group 3 (missing data)	10,477	(33.9)	2559	(24.4)	1233	(11.8)	6685	(63.8)	0.47	(0.44, 0.49)	0.81	(0.73, 0.90)
No childhood vaccination	278	(0.9)	134	(48.2)	69	(24.8)	75	(27.0)				
Childhood vaccination	24,073	(78.0)	8726	(36.2)	4338	(18.0)	11,009	(45.7)	0.44	(0.34, 0.57)	0.42	(0.32, 0.55)
Missing childhood vaccination	6531	(21.1)	1249	(19.1)	450	(6.9)	4832	(74.0)	0.13	(0.10, 0.17)	0.14	(0.11, 0.18)

**Table 2:** Cervical Screening uptake according to characteristics of women in the cohort.

Variable	Number (% for each variable)		Screened (%)		Not screened (%)		Univariate odds ratio (95% CI)		Adjusted odds ratio (95% CI)	
Quintile 1 (least deprived)	4093	(13.3)	2052	(50.1)	2041	(49.9)				
Quintile 2	4323	(14.0)	2188	(50.6)	2135	(49.4)	1.019	(0.936, 1.110)	1.038	(0.952, 1.132)
Quintile 3	5737	(18.6)	2645	(46.1)	3092	(53.9)	0.851	(0.785, 0.922)	0.942	(0.868, 1.023)
Quintile 4	6940	(22.5)	3239	(46.7)	3701	(53.3)	0.870	(0.806, 0.940)	1.026	(0.947, 1.111)
Quintile 5 (most deprived)	9789	(31.7)	4040	(41.3)	5749	(58.7)	0.699	(0.650, 0.752)	0.911	(0.844, 0.984)
Maternal age group 1 (25–29 years)	7576	(24.5)	3982	(52.6)	3594	(47.4)				
Maternal age group 2 (under 20 years)	2163	(7.0)	1075	(49.7)	1088	(50.3)	0.892	(0.810, 0.981)	0.964	(0.875, 1.062)
Maternal age group 3 (20–24 years)	6313	(20.4)	3284	(52.0)	3029	(48.0)	0.979	(0.915, 1.046)	1.015	(0.949, 1.086)
Maternal age group 4 (30–34 years)	4093	(13.3)	2050	(50.1)	2043	(49.9)	0.906	(0.839, 0.977)	0.887	(0.821, 0.958)
Maternal age group 5 (35+ years)	1578	(5.1)	755	(47.8)	823	(52.2)	0.828	(0.743, 0.923)	0.831	(0.745, 0.928)
Maternal age group 6 (missing data)	9159	(29.7)	3018	(33.0)	6141	(67.0)	0.444	(0.417, 0.472)	0.702	(0.617, 0.799)
Gestational age group 1 (37+ weeks)	19,187	(62.1)	9910	(51.6)	9277	(48.4)				
Gestational age group 2 (under 36 weeks)	1218	(3.9)	557	(45.7)	661	(54.3)	0.789	(0.702, 0.886)	0.799	(0.710, 0.898)
Gestational age group 3 (missing data)	10,477	(33.9)	3697	(35.3)	6780	(64.7)	0.510	(0.486, 0.536)	1.005	(0.906, 1.114)
No childhood vaccination	278	(0.9)	110	(39.6)	168	(60.4)				
Childhood vaccination	24,073	(78.0)	12,178	(50.6)	11,895	(49.4)	1.564	(1.228, 1.991)	1.415	(1.105, 1.813)
Missing childhood vaccination	6531	(21.1)	1876	(28.7)	4655	(71.3)	0.616	(0.481, 0.787)	0.840	(0.654, 1.078)
Not HPV vaccinated	15,916	(51.5)	6158	(38.7)	9758	(61.3)	0.512	(0.487, 0.539)	0.580	(0.551, 0.611)
Partial HPV vaccinated	4857	(15.7)	2427	(50.0)	2430	(50.0)	0.811	(0.757, 0.869)	0.789	(0.737, 0.846)
Full HPV vaccinated	10,109	(32.7)	5579	(55.2)	4530	(44.8)				

**Table 3:** Cervical Screening results according to characteristics of women in the cohort.

Variable	Number (% for each variable)		Abnormal (%)		Not abnormal (%)		Univariate odds ratio (95% CI)		Adjusted odds ratio (95% CI)	
Quintile 1 (least deprived)	2052	(14.5)	318	(15.5)	1734	(84.5)				
Quintile 2	2188	(15.4)	344	(15.7)	1844	(84.3)	1.02	(0.86, 1.20)	1.01	(0.86, 1.20)
Quintile 3	2645	(18.7)	378	(14.3)	2267	(85.7)	0.91	(0.773, 1.07)	0.91	(0.77, 1.07)
Quintile 4	3239	(22.9)	507	(15.7)	2732	(84.3)	1.01	(0.87, 1.18)	1.01	(0.87, 1.18)
Quintile 5 (most deprived)	4040	(28.5)	633	(15.7)	3407	(84.3)	1.01	(0.88, 1.17)	1.02	(0.87, 1.18)
Maternal age group 1 (25–29 years)	3982	(28.1)	634	(15.9)	3348	(84.1)				
Maternal age group 2 (under 20 years)	1075	(7.6)	184	(17.1)	891	(82.9)	1.09	(0.91, 1.31)	1.06	(0.88, 1.27)
Maternal age group 3 (20–24 years)	3284	(23.2)	500	(15.2)	2784	(84.8)	0.95	(0.84, 1.08)	0.94	(0.83, 1.07)
Maternal age group 4 (30–34 years)	2050	(14.5)	312	(15.2)	1738	(84.8)	0.95	(0.82, 1.110)	0.96	(0.82, 1.11)
Maternal age group 5 (35+ years)	755	(5.3)	105	(13.9)	650	(86.1)	0.85	(0.68, 1.07)	0.86	(0.69, 1.07)
Maternal age group 6 (missing data)	3018	(21.3)	445	(14.7)	2573	(85.3)	0.91	(0.80, 1.04)	1.05	(0.81, 1.36)
Gestational age group 1 (37+ weeks)	9910	(70.0)	1560	(15.7)	8350	(84.3)				
Gestational age group 2 (under 36 weeks)	557	(3.9)	72	(12.9)	485	(87.1)	0.80	(0.617, 1.02)	0.79	(0.62, 1.02)
Gestational age group 3 (missing data)	3697	(26.1)	548	(14.8)	3149	(85.2)	0.93	(0.838, 1.04)	0.95	(0.77, 1.16)
No childhood vaccination	110	(0.8)	19	(17.3)	91	(82.7)				
Childhood vaccination	12,178	(86.0)	1901	(15.6)	10,277	(84.4)	0.89	(0.54, 1.46)	0.86	(0.51, 1.43)
Missing childhood vaccination	1876	(13.2)	260	(13.9)	1616	(86.1)	0.77	(0.46, 1.29)	0.67	(0.40, 1.12)
Not HPV vaccinated	6158	(43.5)	1031	(16.7)	5127	(83.3)	1.24	(1.12, 1.37)	1.27	(1.15, 1.41)
Partial HPV vaccinated	2427	(17.1)	371	(15.3)	2056	(84.7)	1.11	(0.97, 1.27)	1.10	(0.97, 1.26)
Full HPV vaccinated	5579	(39.4)	778	(13.9)	4801	(86.1)				

**Table 4:** Cervical screening participation and detection of abnormalities in each vaccination group, stratified by deprivation quintile.

HPV Vaccinated	QUINTILE	Screened (%)	Abnormal (%)
Not HPV vaccinated	Quintile 1 (least deprived)	712 (42.1)	119 (16.7)
	Quintile 2	806 (41.7)	130 (16.1)
	Quintile 3	1107 (38.9)	167 (15.1)
	Quintile 4	1474 (40.4)	266 (18.0)
	Quintile 5 (most deprived)	2059 (35.5)	349 (16.9)
Partially HPV vaccinated	Quintile 1 (least deprived)	408 (51.8)	61 (15.0)
	Quintile 2	382 (54.0)	61 (16.0)
	Quintile 3	427 (47.4)	64 (15.0)
	Quintile 4	539 (51.4)	82 (15.2)
	Quintile 5 (most deprived)	671 (47.5)	103 (15.4)
Fully HPV vaccinated	Quintile 1 (least deprived)	932 (57.7)	138 (14.8)
	Quintile 2	1000 (59.5)	153 (15.3)
	Quintile 3	1111 (55.7)	147 (13.2)
	Quintile 4	1226 (54.7)	159 (13.0)
	Quintile 5 (most deprived)	1310 (50.8)	181 (13.8)
